# A Novel Tool to Assess Basic Activities of Daily Living in Spanish Preschoolers

**DOI:** 10.3390/children8060496

**Published:** 2021-06-10

**Authors:** Sabina Barrios-Fernandez, Margarita Gozalo, Andres Garcia-Gomez, Jorge Carlos-Vivas, Dulce Romero-Ayuso

**Affiliations:** 1 Medical-Surgical Therapeutics Department, University of Extremadura, 10003 Cáceres, Spain; 2 Psychology and Anthropology Department, University of Extremadura, 10003 Cáceres, Spain; 3 Education Sciences Department, University of Extremadura, 10003 Cáceres, Spain; agarcil9@unex.es; 4 Health, Economy, Motricity and Education Research Group (HEME), Faculty of Sport Sciences, University of Extremadura, 10003 Cáceres, Spain; jorge.carlosvivas@gmail.com; 5 Physiotherapy Department, University of Granada, 18071 Granada, Spain; dulceromero@ugr.es

**Keywords:** activities of daily living, executive function, child, evaluation, assessment

## Abstract

Background: Basic activities of daily living (BADLs) are those related to self-care. Their performance depends on the development of sensorimotor and cognitive skills, as well as social and environmental aspects. A good performance in BADLs is required for independence and social participation, so they play an important role in early education and early care. We aim to create a tool for BADLs assessment for Spanish preschoolers. Methods: The tool was administered to 303 participants (48.5% boys and 51.5% girls) between three and six years of age. Analyses to find out the factorial structure and internal consistency was carried out. Results: The instrument was composed of 84 items in four scales (eating, personal hygiene, dressing, and daily functioning) with nine factors (oral sensitivity, good manners, manual dexterity, brushing teeth, toilet management, hygiene and grooming, dressing, higher-order and core executive function). Reliability values were from acceptable to preferred (0.74–0.94). Conclusions: The instrument could be useful and shows preliminary good indicators in construct validity and reliability.

## 1. Introduction

### 1.1. Activities of Daily Living Conceptualization and Development

Activities of daily living (ADLs) refers to a group of tasks that every person carries out to be independent. Concretely, basic activities of daily living (BADLs) refer to the ones oriented toward taking care of one’s own body including mobility, feeding, personal hygiene and dressing. Instrumental activities of daily living (IADLs) are activities to support daily life in home and community, often more complex than BADLs, including home management, taking care of others or community mobility [1,2]. BADLs are gradually acquired during childhood, and through practice they become almost automatic, while IADLs are developed through education and practice, with a greater influence on the individual’s life roles [3]. The inability to accomplish ADLs may lead to unsafe conditions, lower participation, caregiver overload and poorer quality of life [4].

ADLs development is related to motor, physical, cognitive and emotional areas [5], but also to practical experience and contextual factors. Thus, their outcomes result from the dynamic intersection of the individual, task/activity and context/environment characteristics [6].

Preschool age is a period of huge growth in ADLs development. Initially, infants are completely dependent on their caregivers in terms of care, even talking of co-occupations. Around 2–4 years, they begin to manage their cutlery, as well as simple clothes. Then, about 5–6 years, most typically developing children perform the most essential tasks/activities included in BADLs, although they usually need assistance from their caregivers for safety reasons or to initiate them, according to their parenting styles and socio-cultural factors. Setting milestones by age about BADLs acquisition is complex, due to the numerous factors that influence them. Table 1 displays some reference examples [7,8].

However, children with neurodevelopmental disorders [11] are often unable to reach these milestones, showing significant challenges, poorer outcomes, delays, and impairments compared to their typical development peers [12,13,14,15].

### 1.2. Underlying Factors in Activities of Daily Living

Several factors are important for proper ADLs performance. On the one hand, brain maturation-associated internal factors, especially to the prefrontal lobe, include processes such as perception, memory, or executive function (EF) [16,17]. EF are a set of cognitive skills necessary for goal-oriented behavior. There is some agreement on considering inhibition and interference control, working memory, and cognitive flexibility as core sub-processes [18,19,20]. Inhibition means being able to control one’s attention, thoughts, emotions, or behaviour, suppressing other stimuli. Working memory let us to briefly maintain information while performing other operations. Cognitive flexibility refers to being able to switch between thoughts or actions depending on the demands of the context [21,22,23]. From core EFs, higher-order EFs are built, including planning (choosing steps to reach a goal), reasoning, and problem-solving [22,24]. Regarding its development, a first phase happens during the first three years of life, where basic skills emerge, and a second one between the third and the fifth years, when different sub-processes begin to coordinate achieving adaptive goals [20,25]. Thus, EF is essential for all ADLs and to succeed in any daily task [26,27,28,29,30].

On the other hand, social and contextual factors are also essentials for ADLs development, including family and school. During childhood, caregivers must provide opportunities for children to practice ADLs in their communities, encouraging their social participation. This repeated practice promotes the establishment of occupational roles and routines, transmitting cultural values to the child [3,31]. Parenting styles are also relevant, considering that democratic styles are associated with greater independence, while overprotection, overcontrol, persistence in performance, or excessive permissiveness negatively affect children’s mental health and sense of competence [32].

### 1.3. Activities of Daily Living in Early Education and Early Intervention Services

In addition to home and community settings, there are two other contexts in which monitoring ADLs development is essential: at school and, when signs of dysfunction are detected in early intervention services. Regarding school, in Spain, preschool education is divided into two stages: 0–3 years, and 3–6 years. Every stage has its own goals, contents, and evaluation criteria. Both stages are structured in three main areas: environment knowledge, languages, and self-knowledge and functional independence [33,34].

Table 2 shows some of the closest contents related to ADLs, including aspects related to EF required for successful performance.

Monitoring children’s development is critical so that appropriate actions can be undertaken as early as possible; either through educational adjustments or referring to early care services [35,36]. These services aim to respond to temporary or permanent needs presented by children with developmental disorders or at risk [37], and in planning and carrying out interdisciplinary interventions.

In early education, ADLs performance is assessed by teachers, mainly through students’ behaviours observation. Families play an unquestionable role in children’s education [38], so teachers must obtain information about ADLs performance of their students in natural environments through their caregivers [34]. Therefore, observational tools or questionnaires completed by caregivers seem to be an interesting tool [39]. They can also be useful for early care therapists since, although therapists commonly work in clinical settings, they also need to collect information about children’s performance in their natural environments [10,40,41,42,43].

### 1.4. Assessment of Activities of Daily Living in Preschoolers

Several observational tools can be considered to assess ADLs in children from three to six years: The Vineland Adaptive Behavior Scales [44,45], The Adaptive Behavior Assessment System [46,47], The Checklist of Adaptive Living Skills [48], The Inventory for Client and Agency Planning [49], The Pediatric Evaluation of Disability Inventory-Computer Adaptive Test [50], The Battelle Developmental Inventory [51], or the Merrill-Palmer-Revised Scales of Development [52]. However, these instruments present limitations to be applied during the ADL’s evaluation process: (1) some of them are not focused on ADLs construct, but on the concept of adaptive behaviour. It can be problematic as adaptive behaviour is not synonymous with ADL, including different domains and giving more or less weight to ADLs according to authors’ points of view [53]; (2) some of them do not cover the full range of BADLs; while (3) others are translated into Spanish but, to our best knowledge, without performing a cultural adaptation process.

### 1.5. Aim

This study aims to present the psychometric properties (construct validity and reliability) of a tool to measure BADLs performance in typically developing Spanish preschoolers aged 3–6 years. We hope this tool will (1) help to characterize the BADLs performance of typically developing children serving as a screening instrument and (2) will be useful to detect deviations from normality in the BADLs development of children with neurodevelopmental disorder diagnoses, helping professionals in early education and early care services.

## 2. Materials and Methods

### 2.1. Study Design

Participants were recruited through schools and social events in Extremadura (Spain). Furthermore, a convenience clinical sample of 11 participants with autism spectrum disorders (ASD) aged 3–6 years was included to analyze the classification ability of the questionnaire.

### 2.2. Participants

Three-hundred and three preschoolers with typical development, aged from 3 to 6 years (3 years = 13.2%; 4 years = 26.1%; 5 years = 32%; and 6 years = 28.7%), participated in the study. The sample was composed of 147 boys (48.5%) and 157 girls (51.5%). All participants provided written informed consent before starting data collection. To be included in the study, participants need to meet the following eligibility criteria: (1) age between 3 and 6 years, (2) attend to ordinary schools, (3) no present disorders according to the Diagnostic and Statistical Manual of Mental Disorders (DSM-5) [11], and (4) provide informed consent.

### 2.3. Instruments and Procedure

#### 2.3.1. Creation of the Basic Activities of Daily Living Assessment in Preschoolers

**Figure 1:**
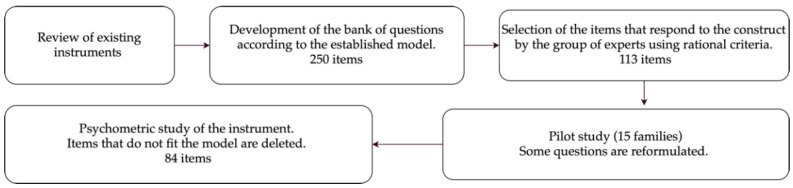
The Basic Activities of Daily Living Assessment in Preschoolers (BADL-P) creation process.

A group of five experts from the clinical field (occupational therapists, and specialists in developmental psychology and neurodevelopmental disorders), selected for their experience in childcare (clinical and educational) and development of psychological tests, were recruited. Initially, an exhaustive review of the available instruments assessing children development, sensory integration, cognitive assessments, and ADLs was carried out. A rational criterion was followed for the selection of behaviours represented in most of the instruments (achieved or in process). The selected items were classified, developing a pool of 250 items. An operational proposal for the different dimensions was submitted to the experts’ judgement. Thus, the experimental version consisted of 113 items. Subsequently, a pilot study was carried out with the participation of 15 families who were asked to answer the questionnaire, assessing the clarity of each item and allowing them to make proposals about wording. They were also informed about the time required to complete the test. Finally, the relationships between the proposed dimensions were explored and the items that did not fit in the model were removed. Thus, the final version of the tool includes 84 items. Figure 1 shows the steps followed for developing the instrument.

#### 2.3.2. Description of the Basic Activities of Daily Living Assessment in Preschoolers Tool

**Figure 2:**
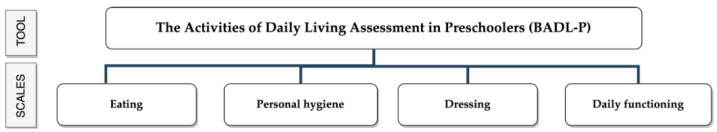
The Basic Activities of Daily Living Assessment in Preschoolers (BADL-P) basic structure.

The Basic Activities of Daily Living Evaluation in Preschoolers (BADL-P), a novel questionnaire created for Spanish preschoolers during this study, was used. The BADL-P included 84 items, distributed in 4 scales with 9 factors that provide a theoretical model to support the instrument. Eating, personal hygiene and dressing are BADLs themselves, as explained, while the daily functioning scale includes information about cognitive skills critical for good BADLs performance (Figure 2).

Most of the items are written in positive form, and those in negative were recoded. This instrument must be completed by interviewing main caregivers. Response options for every item are always, sometimes, never, or not known/no opportunity. Therapists or educators must obtain evidence that caregivers’ answers are as close to reality as possible.

### 2.4. Ethical Approval

The protocol followed in this study adhered to the updates of the Declaration of Helsinki [54], and it was approved by the Committee on Biomedical Ethics of the University of Extremadura (198/2019).

### 2.5. Statistics

Microsoft Office^TM^ Excel v.16 (Redmond, WA, USA: Microsoft Corporation), FACTOR v.10.10.02 (Tarragona, Spain, ESP: Rovira i Virgili University) and IBM^TM^ SPSS v.25 (IBM Corporation, Armonk, NY, USA) were used for data analysis. A semiconfirmatory factor analysis (SCFA) was carried out, that is considered appropriate to prevent errors included in the “Little Jiffy” approach [55,56]. FACTOR performs at the same time an exploratory analysis offering goodness-of-fit indicators, so an additional confirmatory factor analysis is not necessary [57,58,59,60].

Considering the ordinal nature of the data, polychoric correlations using the robust unweighted least squares method with oblique rotation were employed. The Kaiser-Meyer-Olkin (KMO) and Bartlett’s sphericity tests were used as indices of sampling adequacy [61,62]. Due to the comprehensive nature of the tool, which is intended to be used as a developmental scale to monitor BADLs acquisition, and in the absence of cross-loadings, some items with loadings above 0.30 have been included [63].

To assess the goodness-of-fit, we used the chi-squared probability setting as appropriate non-significant values (*p* > 0.05); the comparative fit index (CFI) and the non-normed fit index (NNFI); the root mean square error of approximation (RMSEA); and the root mean square of residuals (RMSR) [62,64].

Ordinal alpha was used to find out the internal consistency of the tool. It represents an alternative to Cronbach’s Alpha for ordinal items, being >0.70 values considered as acceptable and >0.80 preferred [65,66].

As external validity criteria, descriptive and contrast results are provided according to sociodemographic characteristics of the sample. Additionally, preliminary data on the classification ability of the questionnaire are presented through the analysis of the receiver operating characteristic (ROC curves), comparing with a sample of 11 subjects with ASD in addition to the sample of 303 typically developing participants.

## 3. Results

### 3.1. Item Analysis and Internal Structure of the Questionnaire

**Figure 3:**
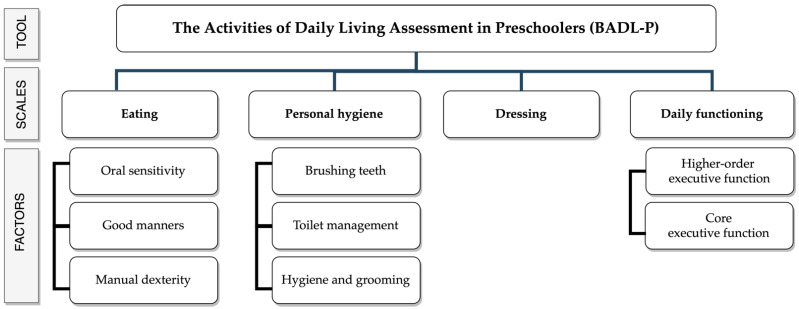
The Basic Activities of Daily Living Assessment in Preschoolers (BADL-P) complete structure.

The BADL-P study version was initially composed of 113 items. After performing the analysis, 29 items were deleted, so the final version was finally formed by 84 items distributed in four scales with nine factors (Figure 3). The instrument is created in Spanish (Supplementary Material), but items are provided in English to facilitate the reading of the paper.

The factor structure of the resulting dimensions and the factor loading of each item are presented below.

#### 3.1.1. Eating Scale

A KMO value of 0.68, and Bartlett’s test, *p* < 0.001 were both good enough to carry out the SCFA. However, 12 items did not reach <0.30 so 16 items formed the final version of the scale.

Eating refers to all the tasks or activities that help in manipulating, keeping food or fluids in the mouth and swallowing [2]. We found an interpretable solution with three factors which explores: (1) items related to sensory integration, (2) items associated with social, educational, and cultural behaviours that must be learnt to be considered nicely behaved during mealtime, and (3) items about hand skills with food, fluids, cutlery, or containers to perform self-feeding (Table 3).

#### 3.1.2. Personal Hygiene Scale

A KMO value of 0.903 and a *p* < 0.001 for Bartlett’s test were found. Initially, 36 items formed the scale, but six items were deleted. Thus, 30 items were maintained.

Personal hygiene refers to obtain and use toileting supplies to get or keep clean, including toileting needs, brushing, washing up, bathing and grooming [2]. We got an interpretable solution with three factors: (1) all the items related to brushing teeth, (2) the ones related to toileting needs, and (3) the rest of personal hygiene and grooming activities (Table 4).

#### 3.1.3. Dressing Scale

A KMO value of 0.952 and a *p* < 0.001 for Bartlett’s test were obtained. The scale had 30 items, but nine items were deleted, so 21 items form this scale.

Dressing refers to being able to select clothes, shoes, and accessories, putting them on and taking them off, and getting dressed and undressed in the right way [2]. This scale is formed only by one factor, as presented in Table 5.

#### 3.1.4. Daily Functioning Scale

A KMO value of 0.737 and a *p* < 0.001 for Bartlett’s test were found. Only 2 items were deleted, so the final version got 17 items on this scale.

This scale joins cognitive aspects that influence BADLs performance, and it is composed of two factors (Table 6): higher-order EF (eight items) and core EF (nine items).

### 3.2. Correlations between Factors

Table 7 provides correlations between the different factors of every scale. All BADLs factors are related to each other. Likewise, EF is related to all BADLs except oral sensitivity. Thus, oral sensitivity seems to function independently, and it is only weakly and negatively related to core EF.

### 3.3. Goodness-of-Fit Indices

Table 8 shows that all the indices, calculated with FACTOR software, are acceptable.

### 3.4. Reliability

Ordinal alpha (Table 9) was used to find out the internal consistency of the BADL-P. Results are acceptable (>0.70) or preferred (>0.80).

### 3.5. Results According to Sociometric Variables and Questionnaire Structure

Table 10 shows descriptive and contrast statistics referring to the participants’ scores considering sex (boys and girls). Significant differences of moderate magnitude according to sex in the toilet management dimension (*p* < 0.04; d > 0.56), with differences in favour of the girls’ group, were observed. Moreover, significant differences of large magnitude are observed in the core EF (*p* < 0.001; d > 1.22) in favour of girls.

Table 11 describe descriptive and contrast statistics after grouping participants according to the age of the two stages within early childhood education (3–4 and 5–6 years). Participants’ scores indicate significant and high magnitude differences in almost all the dimensions, except in oral sensitivity (*p* < 0.22; *d* > 0.19) and core EF (*p* < 0.001; *d* > 1.22). These findings indicate a strong effect of age on the acquisition of BADLs (Table 11).

### 3.6. The Basic Activities of Daily Living Assessment in Preschoolers Discrimination Ability between Typically Developing Participants and a Sample of ASD Participants

Although this manuscript presents the preschool version of our tool in typically developing children, our goal is that it can be used with children with neurodevelopmental disorders. Thus, a preliminary comparison of 11 typical children compared to 11 children with ASD (not included in our study sample) is presented to test the ability of the BADL-P to discriminate between performance on ADLs between typical development and ASD.

**Figure 4:**
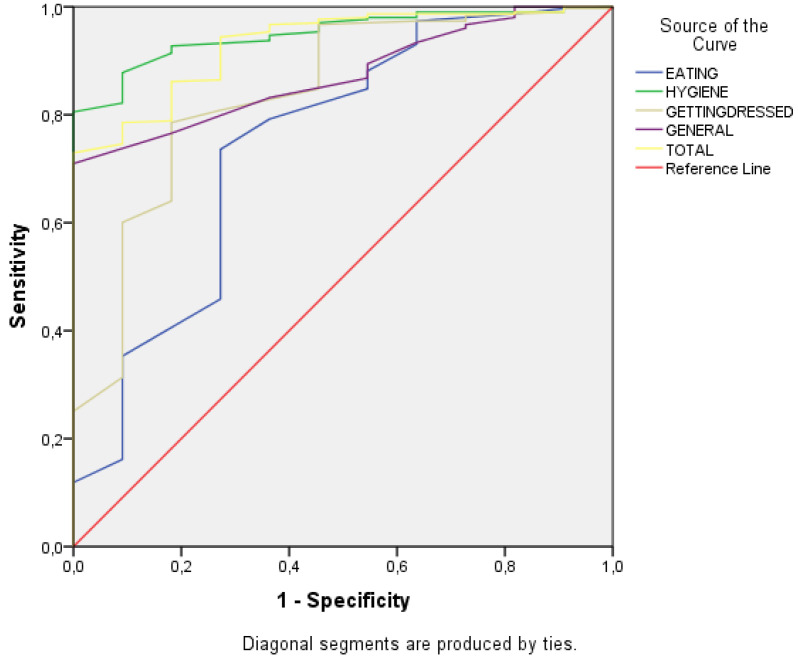
Graphical representation from the receiver operating characteristic (ROC curves).

As illustrated in Figure 4 and Table 12, the area under the curve (AUC) shows that the tool can classify beyond chance between typically developing participants and participants with ASD (*p* < 0.00). The ability to classify between the two groups of greater magnitude is related to the personal hygiene scale and the total score of the questionnaire.

## 4. Discussion

### 4.1. The Basic Activities of Daily Living Assessment in Preschoolers Theoretical Model

This study presents the BADL-P theoretical model, a novel tool for Spanish children between 3–6 years, with good psychometric properties according to preliminary data provided, practical and useful for both, early school educators and early care services educators and therapists. It was initially composed of 113 items, and after the study, they were reduced to 84. The model is divided into four scales: eating, personal hygiene, dressing, and daily functioning scales. The last scale offers a screening of cognitive factors which may influence during ADLs performance. In the eating scale, we got a structure with three factors and 16 items: oral sensitivity (three items), good manners (seven items) and manual dexterity (six items). In the personal hygiene scale, three factors and 30 items: brushing teeth (six items), toileting management (nine items) and hygiene and grooming (15 items). The dressing scale is composed of only one factor with 21 items. Finally, the daily functioning scale is composed of two factors and 17 items: higher-order EF (eight items) and core EF (nine items) during ADLs performance.

In our previous study [68], we presented the scholar version for children between six and 12 years (ADL-E). It was formed by a total of 84 quantitative items and six additional qualitative items only for girls about menstruation management. All items were distributed in the same four scales, but with different factors that outlined the progressive specialization in the BADLs from birth through lifetime. Thus, comparing the preschool version (BADL-P) with the school version (ADL-E), we observe how the dimensions are gradually expanded, as the skills are subdivided as the children grow up, which presents an indicator of validity [17,69].

As observed in Table 7, which shows correlations between factors, core EF, is closely related to BADLs performance. Thus, it is important to determine how problems in both of them could be affecting BADLs performance [30,70,71]. Therefore, our tool might help clinicians to determine whether a more in-depth assessment in one or another direction is needed.

### 4.2. The Basic Activities of Daily Living Assessment in Preschoolers and Other Tools

As exposed, ADLs performance is influenced by both internal and external factors. Occupational development is the result of the dynamic interaction of person, activity and environment [6]. In Spain, some instruments for measuring BADLs are available, but as previously exposed, they present some limitations. On the one hand, sometimes occupational assessment is inferred from instruments that assess adaptive behaviour, described as suitable behaviours for independent living. However, this concept includes some or other areas or activities depending on the classification consulted. For example, Kamphaus (1987) talks about physical/motor, self-help/independence, interpersonal/social, cognitive/communication and responsibility. Meanwhile, Widaman et al. (1993) mention cognitive competence, social competence, social maladaptation, and personal maladaptation [53]. On the other hand, ADLs conceptualization has reached a huge agreement [2,72,73]. The BADL-P structure is focused on ADLs, which is concrete and unambiguous. Some of the most widely used instruments based on adaptive behaviour concept, mix BADLs performance with other social aspects, not covering the BADLs full range of activities, or being their items divided into different sections or categories not according to their nature. For example, the Battelle Developmental Inventory [51], or the Inventory for Client and Agency Planning [49] include few items on BADLs performance. In the Adaptive Behavior Assessment System II [74], the items are divided into different sections which make them difficult to understand. It is also important to note that some tools, such as the Carolina Curriculum [75] or the Merrill–Palmer Revised Scales [52], do not cover the full preschooler stage. Moreover, many of these instruments are available in Spanish, but we have not found information about their validation; e.g., the Pediatric Evaluation of Disability Inventory [76] or the Vineland Adaptive Behavior Scales II [44], with the risk of not being culturally adapted.

### 4.3. The Basic Activities of Daily Living Assessment in Preschoolers Psychometric Properties

We have created our instrument after performing an exhaustive review, using an experts’ group, carrying out a pilot study with families, conducting the study itself and carrying out the corresponding statistical analysis. In relation to statistics, we have carried out the factorial analysis with FACTOR software, which can perform SCFA what means that while performing an exploratory factor analysis shows goodness-of-fit indices to prove if the factorial solution offers a suitable adjustment. The SCFA has been used to validate instruments in natural [77,78], social [79,80,81,82,83,84,85], and health sciences [30,86,87]. Thus, it is a widely contrasted procedure.

As reflected in results section, the goodness-of-fit indices related to the construct validity of the test structure are highly adequate. The descriptive and contrast statistics indicate significant differences related to the two age groups analyzed (2–4 and 5–6 years). It demonstrates that ADLs performance is acquired throughout development and our instrument seems sensitive for analyzing this progression. Furthermore, construct validity is reinforced by additional data on the instrument’s ability to classify between typical participants and a small group of ASD individuals. ROC curves show the instrument’s ability to discriminate adequately on some of the dimensions (Hygiene = 0.95 and Total = 0.93). As it can be noticed, the scores that most discriminate between typical development and ASD preschoolers are the personal hygiene scale and the total score. Concerning hygiene activities, they have a greater contextual load (noises, smells, visual stimulation by mirrors and reflections) and are longer and more precise than feeding and dressing. Therefore, they have a greater demand of core EF. The reliability index provided by the ordinal alpha [88,89,90,91] shows an acceptable level of internal consistency (α > 0.70). In summary, this preliminary study offers promising indicators of reliability and construct validity.

### 4.4. Limitations and Future Lines

This research has some limitations. We are aware that we need to check the concurrent validity using well-established tools and predictive validity, conducting longitudinal studies to check the capacity of the instrument to detect occupational performance issues development of the children. However, we have not found validated tools that support BADLs construct in Spanish preschoolers. Another limitation is the small sample size of ASD children. This is the reason why we are talking about preliminary results, and we are working to get a larger sample size to increase the power of results.

Several future lines will be developed. In this manuscript, we have presented the BADL-P for preschoolers. In a previous study, the scholar version was tested [68]. We are also interested in exploring the skills required to achieve BADLs during the 0–3 years stage. In this line, IADLs (caring for others, communication management, community mobility, financial and home management, spiritual activities, safety and emergency maintenance, and health management [2]), which are performed at home and in the community, gain importance during adolescence. Thus, it could be interesting to explore an instrument in this scope.

Furthermore, our final goal is testing our tools in children and adolescents with neurodevelopmental disorders diagnosis [11], which includes intellectual disability, ASD, attention deficit and hyperactivity, specific learning, motor, and communication disorders. These children usually have poorer ADLs performance compared with typical development children, and some studies draw different occupational profiles in these individuals [12,13,92,93]. At the stage studied, the development of ADLs is closely linked to maturational processes, in addition to other contextual processes, such as the patterns of parenting in each cultural setting. The translation of the scale into other languages and its adaptation to other cultural contexts will bring greater clarity to the influence of both aspects on the development of ADLs. Although this paper presents preliminary discrimination data with a small sample of ASD subjects, we intend to check the specificity–sensitivity of our tools in clinical samples of children with neurodevelopmental disorders.

Finally, we want to highlight the importance of carrying out practices and policies to support children with disabilities to participate in society, even those with “invisible disabilities” like children with neurodevelopmental disorders. Thus, this project is aligned with objectives 3 (“Good-health and well-being”) and 4 (“Quality education”) of the 2030 Agenda for Sustainable Development [94].

## 5. Conclusions

Based on our preliminary results, we conclude that BADL-P is a practical and easy to use tool with good construct validity and reliability properties for assessing BADLs occupational performance in Spanish preschoolers between three and six years.

Although this tool was developed to test BADLs occupational performance in typical development preschoolers aged from three to six years, preliminary results suggest that this tool could discriminate between typically developed children and their peers with neurodevelopmental disorders. However, future studies with larger sample sizes are needed to increase the power of results.

## Figures and Tables

**Table 1:** Examples of activities of basic daily living milestones in typical children [7,9,10].

Age	Feeding	Personal Hygiene	Getting Dressed
3 years	Uses spoon and fork. Drinks safely.	Turns taps. Handles clothes before the toilet.	Takes off his shoes. Takes off his shirt.
4 years	Uses the napkin. Mature spoon and fork grip.	Washes hands and face. Soaps his body.	Puts on top clothes. Buttons up.
5 years	Cuts with the knife. Eats by himself.	Brushes his teeth. Cleans himself in the toilet.	Puts shoes on the right foot. Dresses unsupervised.
6 years	Spreads with a knife. All skills are improved.	Blows his nose. Washes hands before eating.	Laces shoes. Handles zippers.

**Table 2:** Activities of daily living related contents in the early education Spanish curriculum [33].

Preschool—First Stage	Preschool—Second Stage
Area 1. Awakening of personal identity: • Exploration and identification of the parts of the body, pointing and naming them in activities of daily living such as dressing or personal hygiene. Area 2. Personal well-being and daily life: • Progressive adaptation of one’s biological rhythms to socially established routines. • Identification of basic needs such as thirst, hygiene, sleep, satisfying them independently or asking for help. • Acquisition of basic habits and rules regarding food, cleanliness, resting or clothing, identifying utensils and spaces and using them properly. • Satisfaction from participating in activities of daily living, progressively assuming responsibility. • Confidence in one’s possibilities to solve tasks and overcoming difficulties with help.	Area 1. The body and the image itself: • Identification, regulation, and control of the basic needs of the body. Area 3. Activities of daily living: • Performing activities of daily living with progressive independence and the creation of habits. • Initiative, organization, planning, attention, constancy, and regulation skills while performing activities of daily living. Area 4. Personal care and health: • Actions to improve health and well-being for oneself and others. • Healthy habits: body hygiene, food and resting. • Appropriate use of spaces and utensils. • Preference for a well-groomed appearance. • Collaboration in the maintenance of clean and tidy environments. • Respect for the social rules during meals, resting and hygiene, with progressive initiative in their fulfilment.

**Table 3:** Factorial solution of the Eating scale.

Item	Factorial Weight		
**Factor 1: Oral sensitivity.**			
The child is reluctant to try new foods.	0.820		
The child is unwilling to eat food with some textures.	0.843		
The child shows disgust when certain foods are within his mouth.	0.500		
**Factor 2: Good manners.**			
The child tests the food carefully to check its temperature.		0.394	
The child chews with his mouth closed.		0.508	
The child chews food until crushed before swallowing.		0.343	
The child maintains a proper posture during mealtime.		0.685	
The child keeps seated at the table during mealtime.		0.774	
The child uses napkins properly.		0.417	
The child tries to maintain good manners during mealtime.		0.676	
**Factor 3: Manual dexterity while eating.**			
The child can open wrappers.			0.380
The child uses tools to open containers.			0.450
The child uses a knife to spread.			0.747
The child uses a knife to cut food.			0.884
The child uses several cutleries in a coordinated way.			0.792
The child can serve food from a bowl or tray.			0.652

**Table 4:** Factorial solution of the Personal hygiene scale.

Item	Factorial Weight		
**Factor 1: Brushing teeth.**			
The child brushes his teeth after eating without being told by an adult.	0.545		
The child brushes for at least one minute.	0.839		
The child brushes most or all areas of his mouth.	0.836		
The child spits into the wash when brushing his teeth.	0.808		
The child checks there are no traces of paste left in his mouth or face.	0.493		
The child leaves the sink clean and picks up everything after brushing.	0.453		
**Factor 2: Toilet management.**			
The child stays poopless at night.		0.688	
The child stays dry at night, without peeing.		0.510	
The child keeps clean during the day, without pooping himself.		0.726	
The child keeps dry during the day, without peeing himself.		0.746	
The child communicates his need to go to the bathroom.		0.713	
The child acceptably gets clean with toilet paper.		0.384	
The child can lower or raise his clothes to use the toilet.		0.449	
The child lowers the lid and pulls the chain.		0.431	
The child cares about his privacy.		0.313	
**Factor 3: Hygiene and grooming.**			
The child collaborates using cologne or moisturizer.			0.359
The child keeps his nails clean.			0.408
The child brushes his hair.			0.579
The child checks his appearance before leaving home.			0.479
The child is aware when he needs to wipe his nose.			0.540
The child blows his nose.			0.468
The child checks and adjusts the water temperature			0.575
The child when washing his hands, spreads soap and water in his hands.			0.540
The child when washing his hands, uses an adequate amount of soap.			0.519
The child when washing his hands, wipes himself completely dry.			0.467
The child washes his face.			0.677
In the shower, soaps up all over the body.			0.848
In the shower, rinses until all foam is removed.			0.835
In the shower, uses the towel until is relatively dry.			0.714
In the shower, lathers his hair in an acceptable way.			0.635

**Table 5:** Factorial solution of the Dressing scale.

Item	Factorial Weight
The child makes sure that the label of the clothes is in the right place.	0.566
The child put. his socks properly.	0.736
The child puts footwear on his feet.	0.699
The child places a shoe on the right foot.	0.657
The child removes shoes with fasteners.	0.320
The child removes simple garments without closures.	0.538
The child undresses completely, including using zippers on garments.	0.717
The child takes off his clothes, leaving them on the right side.	0.523
The child puts on a coat or an open garment.	0.624
The child puts on stretching pants.	0.733
The child puts on a T-shirt or an upper garment.	0.738
The child gets dressed without help (not including closures).	0.838
The child puts on accessories.	0.518
The child clasps snap buttons.	0.691
The child zips up and down.	0.648
The child zips clothes up.	0.693
The child can unbutton.	0.756
The child opens buttons.	0.775
The child undoes his shoes’ lacing.	0.542
The child ties a knot in his shoes.	0.499
The child gets dressed without help (closures and accessories).	0.817

**Table 6:** Factorial solution of the Daily functioning scale.

Item	Factorial Weight	
**Factor 1: Higher-order executive function.**		
The child begins his activities of daily living in a reasonable time from the adult’s direction.	0.517	
The child can perform his activities of daily living without the help of an adult.	0.554	
The child persists in their activities of daily living although he finds difficulties.	0.306	
The child finishes his activities of daily living at an appropriate time.	0.521	
The child becomes aware of the mistakes he makes in his activities.	0.559	
The child tries to solve problems while performing an activity.	0.732	
The child performs his daily activities without unnecessary stops.	0.642	
The child performs his daily activities in a logical order.	0.649	
**Factor 2: Core executive function.**		
The child gets frustrated quickly when cannot perform an activity.		0.428
The child has more tantrums than expected for his age.		0.459
The child has difficulties to get adapted to changes in the environment.		0.533
The child has difficulties to adapt changes in his routine.		0.622
The child has difficulties moving from one activity to move on to another.		0.506
The child often leaves his activities of daily living unfinished.		0.344
The child loses his attention performing his activities if there is some noise.		0.555
The child spins or rocks excessively, making it difficult to do his activities.		0.654
The child does not perform his activities properly due to excessive movement.		0.546

**Table 7:** Correlations between the BADL-P factors.

	Eating Scale			Personal Hygiene Scale			Dressing Scale	Daily Functioning
	Oral Sensitivity	Good Manners	Manual Dexterity	Brushing Teeth	Toilet Management	Hygiene	Dressing	Higher-Order EF
**Good Manners**	−0.07							
**Manual Dexterity**	−0.00	0.18 **						
**Brushing teeth**	−0.03	0.28 **	0.40 **					
**Toilet management**	−0.01	0.23 **	0.30 **	0.42 **				
**General hygiene**	−0.01	0.39 **	0.46 **	0.48 **	0.49 **			
**Dressing**	−0.04	0.30 **	0.50 **	0.42 **	0.43 **	0.63 **		
**Higher-order EF**	−0.02	0.49 **	0.32 **	0.39 **	0.35 **	0.44 **	0.47 **	
**Core EF**	−0.14 *	0.25 **	−0.05 **	0.06	0.09 **	0.05	0.07	0.19 **

* Significant correlation for *p* < 0.05 ** Significant correlation for *p* < 0.01.

**Table 8:** BADL-P goodness-of-fit indices.

Indices	Cut-off	Eating Scale	Personal Hygiene Scale	Dressing Scale	Daily Functioning Scale
Chi-squared probability *p* (χ^2^)	>0.05	0.000	0.000	0.000	0.009
CFI	>0.90	0.982	0.982	0.987	0.975
NNFI	>0.90	0.972	0.986	0.988	0.981
RMSEA	<0.06	0.039	0.039	0.050	0.034
RMSR	<0.08	0.060	0.073	0.083	0.069

CFI = Comparative fit index; NNFI = non-normed fit index; RMSEA = Root mean square error of approximation; RMSR = Root mean square of residuals.

**Table 9:** BADL-P Internal consistency.

Eating Scale			Personal Hygiene Scale			Dressing Scale	Daily Functioning Scale	
**Manual Dexterity** **Factor**	**Good Manners** **Factor**	**Oral Sensitivity Factor**	**Toilet Management Factor**	**Brushing** **Factor**	**Grooming** **Factor**	**Dressing**	**Higher-Order EF Factor**	**Core EF Factor**
0.81	0.74	0.76	0.80	0.82	0.88	0.94	0.78	0.76

**Table 10:** Descriptive and contrasting statistics by sex group factors.

	Eating Scale			Personal Hygiene Scale			Dressing Scale	Daily Functioning Scale	
Sex	Oral Sensitivity	Good Manners	Manual Dexterity	Brushing	Toilet Management	General Hygiene	Dressing	Higher-Order EF Factor	Core EF Factor
Boys	5.3 ± 1.4	17.7 ± 2.1	10.6 ± 3.1	13.6 ± 2.6	24.3 ± 2.2	35.1 ± 6.0	50.9 ± 7.8	19.4 ± 2.7	12.5 ± 3.2
Girls	5.5 ± 1.3	17.9 ± 2.1	10.5 ± 3.5	13.7 ± 2.7	24.9 ± 2.5	35.7 ± 6.0	52.1 ± 6.9	19.7 ± 2.5	13.7 ± 3.0
*t*	−1.21	−0.48	0.30	−0.14	−2.04	−0.82	−1.51	−0.83	−3.40
*p*	0.22	0.62	0.76	0.88	0.04 *	0.41	0.131	0.40	0.001 **
*d*	0.19	0.12	0.11	0.04	0.56	0.57	1.29	0.25	1.22

*t*: Two-sample *t*-tests; *p*: statistical signification (* 0.05; ** 0.01); *d*: Cohen’d (Small = 0.2; Medium = 0.5; Large = 0.8).

**Table 11:** Descriptive and contrasting statistics by age group factors.

	Eating Scale			Personal Hygiene Scale			Dressing Scale	Daily Functioning Scale	
Years	Oral Sensitivity	Good Manners	Manual Dexterity	Brushing	Toilet Management	General Hygiene	Dressing	Higher-OrderEF Factor	Core EF Factor
3–4	5.4 ± 1.2	17.3 ± 2.2	9.0 ± 2.9	12.6 ± 2.8	23.9 ± 3.1	32.9 ± 6.4	46.9 ± 7.3	18.7 ± 2.2	12.7 ± 2.9
5–6	5.4 ± 1.4	18.1 ± 2.0	11.6 ± 3.1	14.3 ± 2.3	25.0 ± 1.7	37.1 ± 5.1	54.5 ± 5.7	20.1 ± 2.7	13.3 ± 3.3
*t*	−0.01	−3.1	−7.0	−5.4	−3.8	−6.2	−10.1	−4.5	−1.5
*p*	0.992	0.002 **	0.000 **	0.000 **	0.000 **	0.000 **	0.000 **	0.000 **	0.111
*d*	0.00	0.77	2.56	1.72	1.08	4.21	7.67	1.33	0.59

*t*: Two-sample *t*-tests; *p*: statistical signification (* 0.05; ** 0.01); *d*: Cohen’d (Small = 0.2; Medium = 0.5; Large = 0.8).

**Table 12:** Statics from the receiver operating characteristic (ROC curves).

Scales	Typical	ASD	AUC (CI 95%)	*p*	*d*
Eating	33.9 ± 4.4	29.1 ± 5.6	0.74 (0.57–0.91)	0.005	0.936
Hygiene	73.8 ± 9.2	51.6 ± 11	0.95 (0.91–0.98)	0.000	2.327
Dressing	51.5 ± 7.4	39 ± 10.1	0.84 (0.71–0.96)	0.000	1.406
General	32.7 ± 0.4.5	25.7 ± 4.6	0.87 (0.81–0.93)	0.000	1.613
Total	192 ± 20.3	145.5 ± 25.6	0.93 (0.87–0.98)	0.000	2.088

AUC = Area under the curve; CI = Confidence interval; *p* = significance level; *d* = effect size following Cohen criteria [67].

## Data Availability

The datasets used during the current study are available from the corresponding author on reasonable request.

## Supplementary Materials

The following are available online at https://www.mdpi.com/article/10.3390/children8060496/s1. Supplementary materials: The Basic Activities of Daily Living Assessment in Preschoolers (Spanish version).
