# Supplementary material for: A Novel Tool to Assess Basic Activities of Daily Living in Spanish Preschoolers
Source: Children (Basel). 2021 Jun 10;8(6):496. doi: 10.3390/children8060496 (PMC8227419; doi:10.3390/children8060496)
Supplement: Supplementary file 1 [file children-08-00496-s001.zip › children-1238415-supplementary.pdf]

ESCALA: ALIMENTACIÓN

		Siempre	A veces	Nunca	NS/NO
	**Sensibilidad oral.**				
1.	Es reacio/a a probar alimentos nuevos.				
2.	Es reacio/a a comer alimentos de ciertas texturas (purés, sólidos, crujientes…).				
3.	Demuestra asco al tener ciertos alimentos en la boca.				
	**Buenas maneras durante la hora de la comida.**				
4.	Prueba con cuidado la comida para comprobar su temperatura.				
5.	Mastica con la boca cerrada.				
6.	Mastica alimentos hasta triturarlos antes de tragar.				
7.	Mantiene una postura adecuada para comer.				
8.	Se mantiene sentado/a en la mesa el tiempo que dura la comida.				
9.	Utiliza la servilleta cuando se mancha.				
10.	Trata de mantener unos modales adecuados en la mesa.				
	**Destreza manual en alimentación.**				
11.	Es capaz de abrir envoltorios (p.e. bolsa de gusanitos).				
12.	Usa instrumentos para abrir envases (p.e. abrelatas, abridores…).				
13.	Es capaz de untar alimentos con el cuchillo.				
14.	Usa el cuchillo para cortar alimentos.				
15.	Hace un uso combinado y coordinado de varios cubiertos a la vez, (p.e. cuchillo y tenedor).				
16.	Es capaz de servirse alimentos desde un bol o bandeja.				

* NS/NO: No sabe/no oportunidad.

	**Observaciones**

Copyright © 2020 Barrios-Fernández, Gozalo-Delgado, García-Gómez and Romero-Ayuso.

ESCALA: ASEO E HIGIENE PERSONAL		Siempre	A veces	Nunca	NS/NO
	**Cepillado de dientes.**				
1.	Se cepilla los dientes después de comer, sin que un adulto se lo indique.				
2.	Se cepilla durante, al menos, un minuto.				
3.	Se cepilla por la mayoría o por todas las zonas de la boca.				
4.	Se enjuaga y escupe dentro del lavado.				
5.	Tras el cepillado, comprueba en el espejo que no quedan restos de pasta en su boca o cara.				
6.	Deja el lavabo limpio y recoge todo tras cepillarse los dientes.				
	**Control de esfínteres y manejo de las necesidades en el WC.**				
7.	Generalmente, permanece sin hacerse caca de noche.				
8.	Generalmente, permanece seco de noche, sin hacerse pis encima.				
9.	Se mantiene limpio durante el día, sin hacerse caca encima.				
10.	Se mantiene seco/a durante el día, sin hacerse pis encima.				
11.	Indica la necesidad de ir al baño.				
12.	En el baño, se limpia con el papel higiénico de forma aceptable.				
13.	Es capaz de bajar o subir sus prendas para usar el WC.				
14.	Cuando ha acabado de usar el baño, baja la tapa y tira de la cadena.				
15.	En el baño, cuida aspectos de su privacidad (p.e. se desviste y viste dentro del baño).				
	**Higiene personal y acicalamiento.**				
16.	Colabora al echarse colonia o crema hidratante.				
17.	Mantiene limpias sus uñas.				
18.	Se cepilla el cabello.				
19.	Comprueba su aspecto tras vestirse o antes de salir de casa.				
20.	Usa un pañuelo cuando nota que tiene mocos.				
21.	Sabe sonarse los mocos con un pañuelo.				
22.	Es capaz de comprobar y graduar la temperatura del agua.				
23.	Cuando se lava las manos, se da agua y jabón por todas las zonas de las manos.				
24.	Cuando se lava las manos, se echa una cantidad adecuada de jabón.				
25.	Cuando se lava las manos, se seca las manos completamente.				
26.	Es capaz de lavarse la cara.				
27.	En la ducha, se enjabona por todas las partes del cuerpo.				
28.	En la ducha, se enjuaga hasta que elimina toda la espuma.				
29.	En la ducha, se seca con la toalla hasta quedar relativamente seco.				
30.	En la ducha, se enjabona el pelo de manera aceptable.				

* NS/NO: No sabe/no oportunidad.

	**Observaciones**

Copyright © 2020 Barrios-Fernández, Gozalo-Delgado, García-Gómez and Romero-Ayuso.

	ESCALA: VESTIDO	Siempre	A veces	Nunca	NS/NO
1.	Se asegura de que la etiqueta esté por dentro y por detrás antes de ponerse las prendas.				
2.	Es capaz de ponerse los calcetines adecuadamente.				
3.	Es capaz de meterse el calzado en el pie.				
4.	Se pone el zapato en el pie que corresponde.				
5.	Se quita los zapatos con elementos de sujeción como lazadas, hebillas o cremalleras.				
6.	Es capaz de quitarse prendas sencillas que no tengan cierres (p.e. un pantalón de chándal).				
7.	Se desviste completamente, incluyendo los cierres de las prendas.				
8.	Se quita la ropa dejándola del derecho.				
9.	Es capaz de ponerse el abrigo o una prenda abierta.				
10.	Se pone una prenda superior, como una camiseta (no importa si del derecho o del revés).				
11.	Es capaz de vestirse prácticamente sin ayuda (sin incluir cierres de las prendas).				
12.	Se pone complementos (p.e. guantes, bufanda, gorro, etc.).				
13.	Abrocha botones de presión (tipo clic).				
14.	Abre y/o cierra cremalleras.				
15.	Es capaz de unir el pie o cierre de las cremalleras.				
16.	Desabotona botones.				
17.	Abotona botones.				
18.	Deshace la lazada de los zapatos.				
19.	Es capaz de hacer el nudo en sus zapatos.				
20.	Es capaz de hacer la lazada en sus zapatos.				
21.	Es capaz de vestirse prácticamente sin ayuda (incluyendo cierres de las prendas y complementos).				

* NS/NO: No sabe/no oportunidad.

	Observaciones

Copyright © 2020 Barrios-Fernández, Gozalo-Delgado, García-Gómez and Romero-Ayuso.

NOTA: Recuerde, cuando hablamos de Actividades de la Vida Diaria, nos referimos a las relacionadas con alimentación, higiene personal y vestido.

**ESCALA: FUNCIONAMIENTO DIARIO**		Siempre	A veces	Nunca	NS/NO
	**Funciones ejecutivas de orden superior.**				
1.	Inicia sus actividades de la vida diaria en un tiempo prudencial, desde que el adulto se lo indica (p.e. sentarse a la mesa a comer o irse a la ducha).				
2.	En general, es capaz de realizar sus actividades de la vida diaria sin la ayuda del adulto.				
3.	Persiste, trata de mantenerse en actividades de la vida diaria en las que se encuentra con dificultades (p.e. si no le sale la lazada, le cuesta abrocharse un botón, partir un filete, etc.).				
4.	Acaba sus actividades de la vida diaria en un tiempo adecuado, ni demasiado pronto, ni demasiado tarde (p.e. peinarse o lavarse las manos).				
5.	Se da cuenta de los errores que comete en sus actividades de la vida diaria (p.e. si la lazada del zapato le ha quedado flojo).				
6.	Trata de resolver los problemas que se le presentan mientras realiza una actividad de la vida diaria (p.e. si se pone la ropa al revés, se queda sin pasta de dientes, etc.).				
7.	Es capaz de realizar sus actividades de la vida diaria sin parar durante dichas tareas de forma innecesaria.				
8.	Realiza sus actividades de la vida diaria siguiendo sus pasos en un orden lógico (p.e. primero se pone la ropa interior y luego la exterior).				
	**Funciones ejecutivás básicas.**				
9.	Se frustra fácilmente cuando no es capaz de realizar alguna de sus actividades de la vida diaria				
10.	Tiene más rabietas de lo esperable para un niño/a de su edad.				
11.	Le cuesta adaptarse a cambios en su ambiente (p.e. cambiarse de ropa o no desayunar en su tazón favorito).				
12.	Le cuesta asimilar cambios en su rutina (p.e. si normalmente primero se viste y luego desayuna, que un día se haga a la inversa).				
13.	Tiene dificultades para dejar de hacer una actividad y pasar a otra, especialmente si la actividad que está realizando le gusta.				
14.	A menudo deja sus actividades de la vida diaria sin acabar (p.e. cuando se seca las manos, éstas siguen mojadas).				
15.	Pierde la atención si hay algún ruido ambiental durante la realización de sus actividades de la vida diaria (p.e. escucha la aspiradora mientras se lava los dientes).				
16.	En ocasiones, se gira o mece en exceso, perjudicando la ejecución de sus actividades de la vida diaria.				
17.	Desarrolla sus actividades de la vida diaria (vestirse, asearse, alimentarse…) de manera inadecuada por un exceso de movimiento (no para de moverse).				

* NS/NO: No sabe/no oportunidad.

	**Observaciones**

Copyright © 2020 Barrios-Fernández, Gozalo-Delgado, García-Gómez and Romero-Ayuso.
